# Plants Probiotics as a Tool to Produce Highly Functional Fruits: The Case of *Phyllobacterium* and Vitamin C in Strawberries

**DOI:** 10.1371/journal.pone.0122281

**Published:** 2015-04-15

**Authors:** José David Flores-Félix, Luis R. Silva, Lina P. Rivera, Marta Marcos-García, Paula García-Fraile, Eustoquio Martínez-Molina, Pedro F. Mateos, Encarna Velázquez, Paula Andrade, Raúl Rivas

**Affiliations:** 1 Departamento de Microbiología y Genética, Universidad de Salamanca, Salamanca, Spain; 2 REQUIMTE/, Laboratório de Farmacognosia, Departamento de Química, Faculdade de Farmácia, Universidade do Porto, Porto, Portugal; 3 Centro Hispano Luso de Investigaciones Agrarias (CIALE), Universidad de Salamanca, Salamanca, Spain; 4 Unidad Asociada Universidad de Salamanca-CSIC (IRNASA), Salamanca, Spain; University of Malaga-Consejo Superior de Investigaciones Científicas, SPAIN

## Abstract

The increasing interest in the preservation of the environment and the health of consumers is changing production methods and food consumption habits. Functional foods are increasingly demanded by consumers because they contain bioactive compounds involved in health protection. In this sense biofertilization using plant probiotics is a reliable alternative to the use of chemical fertilizers, but there are few studies about the effects of plant probiotics on the yield of functional fruits and, especially, on the content of bioactive compounds. In the present work we reported that a strain of genus *Phyllobacterium* able to produce biofilms and to colonize strawberry roots is able to increase the yield and quality of strawberry plants. In addition, the fruits from plants inoculated with this strain have significantly higher content in vitamin C, one of the most interesting bioactive compounds in strawberries. Therefore the use of selected plant probiotics benefits the environment and human health without agronomical losses, allowing the production of highly functional foods.

## Introduction

The increasing interest in the preservation of the environment and the health of consumers is changing production methods and food consumption habits. Consumers increasingly demand safe functional foods that have beneficial properties for health mainly focused on the protection against carcinogenesis and oxidative processes. The consumption of fresh fruits and vegetables containing bioactive compounds has increased considerably in recent years and many studies have been carried out on the potential benefits of such compounds in different aspects of human health [1]. At the same time, there has been a strong increase in studies addressing the benefits of biofertilization for plants and the environment. In this sense plant promoting rhizobacteria (PGPR) able to colonize the inside of plants tissues are especially interesting [2, 3]. These beneficial microorganisms are plant probiotics [4, 5] and promote the plant growth through different direct mechanisms such as nitrogen fixation, phosphate solubilization, and the production of different compounds such as phytohormones or indirect mechanisms such as the production of siderophores [6]. To achieve both aims, the promotion of plant growth and the benefits for human health, it is necessary to use non-pathogenic microorganisms in biofertilization schemes [7].

*Phyllobacterium* is a genus of non-pathogenic bacteria originally isolated from leaf nodules of some plant families [8] that contains several recently described species associated with plant roots [9] and endophytes of legume nodules [10, 11]. Some strains of this genus are able to fix atmospheric nitrogen [12] and the ability to promote the plant growth has been studied in the type strain of the species *Phyllobacterium brassicacearum* 29-15^T^ (= STM 196^T^), which was originally isolated from the roots of *Brassica napus* [13]. This strain promotes the root growth of *Brassica*, the plant from which it was isolated [13, 14], and in the model plant *Arabidopsis* [15, 16].

Thus, *Phyllobacterium* is a plant probiotic that has been not linked to disease in humans; it is a good candidate as a biofertilizer and is especially interesting for plants whose fruits are consumed raw, in particular those that are considered functional foods such as strawberries [17]. These fruits are rich in dietary fiber and fructose which may help to regulate blood sugar levels and to control calorie intake owing to their satiating effects [18]. Nevertheless, the main interest of strawberries lies in their antioxidant potential linked to the preservation of cardiovascular health [18] and the enhancement of body defenses against oxidative challenges [19]. The antioxidant potential of strawberries is mainly due to their content in vitamin C [18] and the serum content of this vitamin is increased after strawberry intake in health subjects [20, 21]. Therefore, besides their production, it is particularly important to evaluate the effect of plant probiotics on the vitamin C content in strawberry fruits.

The aim of this work was to study the effect of the inoculation of the type strain of *Phyllobacterium endophyticum*, a species isolated from legume nodules [10], on strawberry plants. The results showed that this strain was able to produce biofilms in abiotic and plant surfaces attaching the roots of strawberry plants whose growth as well as their content in nitrogen, phosphorous, potassium, calcium and iron of fruits were increased. Nevertheless, the most relevant result was the increase of the vitamin C content of strawberry fruits that is reported by first time in this study for a bacterial probiotic. Our results showed that the inoculation of strawberries with selected plant probiotic bacteria is not only a beneficial practice for the agriculture and the environment but also for human health.

## Material and Methods

### GFP-labeling of strain PEPV15

The strain PEPV15 was GFP-labeled in order to monitor its presence in the roots of strawberry plants. To label the strain, the plasmid pHC60 [22] was introduced into strain PEPV15 by biparental mating using *E*. *coli* S17.1 [23] as donor strain. For this mating, fresh cultures of donor and recipient strains were mixed on YMA plates [24] and incubated overnight at 28°C. Transconjugants were selected on minimal medium [25] plates supplemented with antibiotic (tetracyclin at 10μg/ml). Transfer of pHC60 to strain PEPV15 afforded bacteria expressing the expected GFP as detected by fluorescence microscopy using a NIKON eclipse 8Oi fluorescence microscope. The recombinant strain was routinely grown at 28°C in TY medium [26] supplemented with tetracycline (10 μg/ml).

### Biofilm production and plant colonization assays

Biofilm formation is an essential step in the colonization of plant roots [27, 28]. The ability of strain PEPV15 to form biofilms on abiotic surfaces has been tested at macro and micro-scale following the protocol described by Wang *et al*. [29] with several modifications. Borosilicate glass tubes were filled with 5 ml of TY medium containing 10 μl of saturated overnight cultures as inocula. After 5 days of incubation at 28°C, when the stationary phase had been reached, the content of each tube was gently removed. The tubes were rinsed with water and stained with 6 ml of 1% crystal violet for 5 min, then they were washed with sterile water, dried and photographied.

The microstructure of the biofilm was observed following the method described by Wetland *et al*. [30] to reveal DNA content, which reflects the growth rate, and was visualized by using acridine orange. For this 25 ml of TY medium were placed in a 50 ml glass tube containing a sterile microscope slide, and this medium was inoculated with 100 μl of a 0.5 OD suspension of the strain PEPV15, using a two-days-old culture in the same medium. The inoculated slides were observed at 5 and 7 days after inoculation. Then, the slide was removed and placed in water three times to remove the bacteria that had not adhered to the slide surface. Following this, the slide was placed in a solution of 40 mg l^-1^ acridine orange in phosphate buffer (pH 7.2) for 30 seconds and one side of the slide was cleaned to allow correct microscopic observation which was carried out using an adequate filter using a NIKON eclipse 8Oi fluorescence microscope.

To analyse the cellulose production YMA plates supplemented with 0,25% of Congo Red were inoculated due to ability of this compound to link β 1–4 bonds (present in cellulose-like polysaccharides) that allows the staining of bacterial colonies of red colour [28]. After seven days, the presence of this colour indicative of cellulose-like polysaccharide production was analyzed in the colonies of strain PEPV15. To confirm cellulose production we followed the methodology used by Robledo *et al*. [28]. The strain PEPV15 was inoculated in 30 ml of YMB liquid medium [24] and incubated in an orbital shaker at 28°C for 5 days at 180 rpm followed by static growth for 2 days at the same temperature. Then 5 ml were recoved from the bottom of the flask and centrifuged at 1500 x g during 5 min. The flocs were then whased with 5 ml of 100 mM PCA buffer pH 5, recovered by centrifugation and resuspended in 5 ml of the same buffer. The suspension was placed in 5mm diameter petri plates and treated with 10 U ml^-1^ cellulase from *Trichoderma viride* (Sigma Co., USA) for 2h at 37°C in an orbital shaker at at 180 rpm. Control without cellulase were incubated in the same conditions.

For plant colonization assays, 30 achenes of *Fragraria x ananassa* var. Camarosa were surface-sterilized by immersion in 70% ethanol for 30 seconds followed by soaking in an aqueous 5% sodium hypochlorite solution for 15 min. The achenes were then washed six times with sterile water, and were germinated in water-agar plates overlaid with Whatman number 1 sterile paper wetted with sterile water. The plates were placed in darkness in a growth chamber at 24°C until the seedling roots were 1–2 cm in lenght.

The GFP-tagged strains were grown for 48h at 28°C on YMA plates and then cells were washed twice and resuspended in sterile water at a final concentration of 10^8^ CFU/ml. The strawberry seedlings were inoculated with 1ml of this suspension.

The seedlings were maintained in the dark and the roots were observed at 5, 7 and 9 days post inoculation using light microscopy and after 14 and 21 days post inoculation using confocal microscopy. In order to remove unbound bacteria, the roots were gently washed three times with sterile distilled water before microscopic observation. Uninoculated roots of strawberry were included in the experiment as negative controls. Fluorescence microscopy was carried out with a Nikon Eclipse 80i, and the excitation of green fluorescent proteins was accomplished using a mercury lamp. Confocal spectral microscopy was performed with a Leica SP5 confocal microscope equipped with a krypton–argon laser using a blue excitation filter (excitation maximum 488 nm; 530-nm long-pass filter). Root cells were stained with 10 μM of propidium iodide (Sigma, USA). Projections were made from adjusted individual channels in the image stacks using Leica software as previously described [31].

### Analysis of plant growth promotion mechanisms in vitro

In this study three mechanisms of *in vitro* plant growth promotion commonly shown by PGPR [6, 7, 32] were analyzed: solubilization of phosphate and siderophore and indole acetic acid production. The solubilization of insoluble phosphate was analysed on YED-P plates containing 2% CaHPO_4_ which were incubated for 15 days at 28°C [32]. Siderophore production was evaluated in M9-CAS-AGAR [33] modified with the addition of a cationic solvent, HDTMA, to stabilize the Fe-CAS complex providing the characteristic colour [34]. Indole acetic acid production was evaluated in JMM medium [35] supplemented with 107mg l^-1^ of tryptophan. After 7 days of incubation the supernatants were recovered by centrifugation at 5000xg and filtered using 0.22 μm Millipore filters (Millipore Co., Amicon, USA). Then 1ml of Salkowsky reagent was added to 2ml of supernatant and the red colour formed was measured by spectrophotometry at 550 nm using an ATI Unicam 8625 Spectrometer (Mattson, USA) [36].

### Growth promotion assays in planta

The ability to promote the plant growth must be confirmed in plant assays and here the ability of PEPV15 to promote plant growth was evaluated on *Fragraria x ananassa* var. Camarosa plants. A total of 15 plants were included in each treatment, control without inoculation and inoculation with strain PEPV15. The frozen seedlings were obtained from a commercial distributor of strawberry plants and were of Canadian origin. They were planted on peat/vermiculite (3:1 in volume) using black plastic trays containing 6 Kg of this mix each one. Six days after planting each plant was inoculated with 100ml of a suspension of the strain PEPV15 containing 1x10^6^ CFU/ml. To obtain this suspension the cells of the strain cultivated on YMA plates for 48h at 28°C were suspended in sterile water.

Plants were irrigated with water from a bottom reservoir every 48 h. The plants were maintained for 3 months in a greenhouse illuminated with natural light in summer (night temperature ranging from 15 to 20°C and day temperatures ranging from 25 to 35°C) with humidity control. At the end of the experiment, stolons, flowers and fruits were counted, fruits were harvested and their fresh and dry weights were measured. The fresh and dry weights were obtained in 25 mature fruits per treatment harvested during the three months of production in the 15 plants from this assay. The analysis of N, P, K, Ca and Mg were performed at the Ionomic Service of CEBAS-CSIC (Spain). Statistical analyses were carried out using StatView program for Macintosh computers. Data were analyzed by one-way analysis of variance, and mean values were compared with Fisher's Protected LSD test (Least Significant Differences) (P≤0.05).

### Analysis of vitamin C

As was stated in the Introduction, the vitamin C is the main antioxidant compound in strawberry fruits and hence we evaluated the effect of the inoculation of strain PEPV15 on the content of this vitamin. For this assay fruits of *Fragraria x ananassa* var. Camarosa from each treatment were collected after the end of microcosmos experiments and they were frozen at -20°C, lyophilized (Labconco Freezone 4.5 apparatus, USA) and ground (mean particle size lower than 910 μm). Then, the fruits were divided into three aliquots that were analyzed separately.

Each ground lyophilized sample (ca 0.3 g), was extracted with 0.01 N H_2_SO_4_ (ca 50 ml) for 30 min under stirring (300 rpm). The aqueous solution was then passed through a Chromabond C18 NEC column (50μm particle size, 60 Å porosity; 10 g sorbent mass/70ml reservoir volume) from Macherey-Nagel (Düren, Germany), previously conditioned with 30 mL of methanol and 70 ml of acid water (pH 2 with HCl). The aqueous extract containing vitamin C (ascorbic acid) was evaporated until dryness under reduced pressure (30°C) and redissolved in 0.001N sulfuric acid (1 ml) for HPLC-UV analysis (20 μl). The separation and quantification of vitamin C was carried out in a system consisting of an analytic HPLC–UV unit (Gilson Inc., Middleton, WI) with an ion exclusion column Nucleogel Ion 300 OA (300 x 7.7 mm; Macherey–Nagel, Düren, Germany), as previously reported by Dopico-García *et al*. [37]. Elution was performed in isocratic mode with H_2_SO_4_ (0.01 N) at a flow rate of 0.2 ml/min. Detection was achieved with a UV detector set at 214 nm [38]. Organic acids quantification was achieved by measuring the absorbance recorded in the chromatograms relative to a standard of vitamin C (Sigma-Aldrich, USA). Data were analyzed by one-way analysis of variance as previously indicated.

## Results

### Biofilm production and colonization of strawberry roots by Phyllobacterium

**Fig 1:**
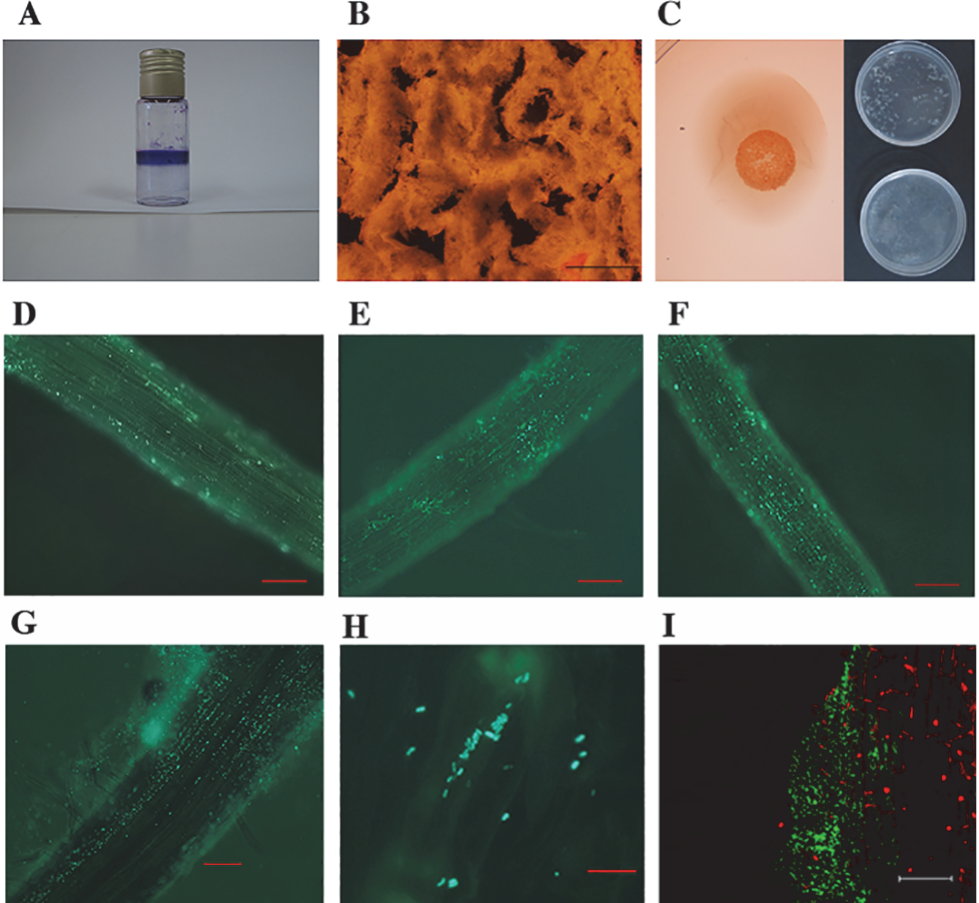
Biofilm formation on abiotic surfaces observed in borosilicate glass tubes with crystal violet (A) and in glass slides with acridin orange (B, bar 500 μm). Cellulose formed in plates containing Congo Red (C, left) and flocs after incubation 2h at 37°C in PCA buffer pH 5 (C, right, up) and and containing 10 U/ml *Trichoderma viride* commercial cellulase (C, right, below). Fluorescence optical micrographs of strawberry seedlings roots 3, 5, 6 and 7 days (D, E; F and G) after inoculation with GFP-tagged cells of PEPV15 strain (D, E, F and G, bar 100 μm, and H, bar 10 μm) and confocal microscopy with epifluorescence using propidium iodide as contrast dye (it stains the plant tissues) after 21 days inoculation (I, bar 67.82 μm). The micrographs show the ability of strain PEPV15 to colonize the roots surfaces of strawberry and the initiation of microcolonies.

Strain PEPV15 was able to produce biofilms in abiotic surfaces (Fig 1A and 1B) and cellulose (Fig 1C). The observation by fluorescence microscopy of GFP-tagged strain PEPV15 inoculated on strawberry seedling roots showed that attachment gradually increased until it peaked at 7 days after inoculation (Fig 1D, 1E, 1F, and 1G). The observation with light microscopy after 7 days of inoculation showed the bacteria firmly attached to root surfaces (Fig 1G) and revealed typical microcolonies of biofilm initiation (Fig 1H). Confocal microscopy at 21 days post inoculation confirmed the colonization of strawberry roots by strain PEPV15 (Fig 1I).

### Plant growth promotion mechanisms

The results of this work showed that strain PEPV15 was able to solubilize moderate amounts phosphate (5mm radius around colonies). The strain grew on the CAS indicator medium where the colonies were surrounded by a yellow-orange halo (3.5 mm radius around colonies) indicative of siderophore production. This strain was able to grow in nitrogen- free liquid medium and in JMM medium supplemented with 170 mg l^-1^ tryptophan producing 19 mg l^-1^ of indole acetic acid; i.e. 10 times more than the amount reported for strain STM 196^T^. Therefore the strain PEPV15 has several direct and indirect *in vitro* plant growth promotion mechanisms indicating that this strain is a good candidate biofertilizer.

### Strawberry growth enhancement by Phyllobacterium

The results of the greenhouse experiments (Table 1) showed that the inoculation with the strain PEPV15 resulted in an increase of several parameters related to plant growth.

**Table 1:** Results from greenhouse experiment in strawberry plants.

Vegetative parameters			Chemical composition		
Treatment	Control	PEPV15		Control	PEPV15
Stolons per plant (± S.E.)	3 (±0.54)^a^	5 (±0.38)^b^	Vitamin C (mg kg^-1^) (± S.E.)	2258.00 (±79.1)^a^	4042.60 (±545.2)^b^
Stolons length (cm) (± S.E.)	44.10 (±2.19)^a^	81.89 (±3.43)^b^	N (%) (± S.E.)	0.94 (±0.01)^a^	1.16 (±0.07)^b^
Flowers per plant (± S.E.)	7 (±1.04)^a^	11 (±1.09)^b^	P (%) (± S.E.)	0.22 (±0.05)^a^	0.31 (±0.06)^b^
Fruits per plant (± S.E.)	3 (±0.44)^a^	5 (±0.45)^b^	K (%) (± S.E.)	1.47 (±0.03)^a^	1.72 (±0.04)^b^
Fresh weight per fruit (g)^¥^ (± S.E.)	11.45 (±0.67)^a^	13.31 (±0.44)^a^	Fe (mg kg^-1^) (± S.E.)	18.94 (±0.04)^a^	24.95(±0.19)^b^
Dry weight per fruit (g)^¥^ (± S.E.)	0.89 (±0.70)^a^	1.01 (±1.23)^b^	Ca (%) (± S.E.)	0.10 (±0.01)^a^	0.19 (±0.01)^b^

Values followed by different letter in each treatment are significantly different from each other at p<0.05. S.E. = Standard Error.

^¥^ Results from 25 fruits per treatment from first to fourth categories.

Strawberry plants have some peculiarities in the sense that they have stolons from which other plants can be obtained and that the plants maintain the production of fruits over more than two months. The number of stolons as well as their length were significantly higher (p-value lower than 0.05) in the plants inoculated with the strain PEPV15, and the plants also produced significantly higher number of flowers and fruits (Table 1). The fruits produced were classified in the commercial categories recognized by the EU (Commission Regulation (EEC) No 899/87) depending on their caliber (extra, first, second and third category, all of which correspond to fruits weighing more than 7g). Although there were not significant differences, the mean fresh weight of the fruits from plants inoculated with strain PEPV15 was higher than that of those from the uninoculated control. These results showed that the strain PEPV15 is a good plant probiotic for strawberries increasing the yield in stolons, flowers and fruits.

Regarding the mineral content of plants, the content of several ions was significantly higher (p-value lower than 0.05) in the fruits from plants treated with strain PEPV15, as can be seen in Table 1. Nitrogen, phosphorous, calcium, potassium and iron contents were increased in the inoculated plants, showing that strain PEPV15 increased not only the yield of strawberry plants but also the quality of their fruits.

### Increase in the vitamin C content in strawberry fruits

The results obtained showed that the vitamin C content in strawberry fruits from plants inoculated with strain PEPV15 was significantly higher (almost double) than in fruits from uninoculated plants (Table 1). Therefore, the inoculation with strain PEPV15 increases the yield, the quality and the functionality of strawberry fruits.

## Discussion

To be considered as plant probiotics, the microorganisms must play a beneficial role in plant development, but when they are endophytic and the plants are to be consumed raw, the microorganisms used in biofertilization must be safe for humans [7]. Many potentially PGPR cannot be considered as useful plant probiotics because they are either pathogenic or closely related to pathogenic species, as discussed by García-Fraile *et al*. [7]. Thus, harmful compounds used in chemical fertilization cannot be replaced by potentially dangerous biofertilizers [39], particularly when they are applied to functional fruits used to used to boost human health. Accordingly, it is necessary to select the plant probiotics among bacteria that have not been related to human diseases, as occurs in the case of the strain PEPV15, which belongs to genus *Phyllobacterium* whose species have so far not been related to human infections.

This strain was able to produce biofilms, a common ability of bacteria living in a self-produced matrix of hydrated extracellular polymeric substances that form their immediate environment and that are mainly polysaccharides, proteins, nucleic acids and lipids [27]. Cellulose is one of components of the biofilm polysaccharides and is an important mechanism for the colonization of roots and others types of surface in the case of rhizobia [28]. In this work we showed that strain PEPV15, phylogenetically close to rhizobia [10], is also able to form biofilms on abiotic surfaces and to produce cellulose. This ability was also seen on the surfaces of strawberry roots where microcolonies of this strain were observed with confocal microscopy.

The ability to colonize strawberry roots, together with the presence of direct and indirect *in vitro* mechanisms of plant growth promotion in strain PEPV15, makes it a good candidate as a probiotic for strawberries. The auxine production (indol acetic acid), the solubilization of phosphate and the nitrogen fixation by the strain PEPV15 may have positive effects in strawberry growth that were confirmed after plant inoculation with this strain since the plants inoculated had more stolons, flowers and fruits than the uninoculated ones. Moreover, in the fruits from inoculated plants the contents of the macroelements nitrogen and phosphorous and the microelements calcium, potassium and iron were higher than in fruits from uninoculated plants. No differences in these parameters were found in other studies after the inoculation with PGPR from the genera *Pseudomonas* or *Bacillus* [40, 41]. Our results, together with those obtained for *P*. *brassicacearum* in other plants [13, 14, 15, 16], confirm that members of genus *Phyllobacterium* are good plant probiotics, increasing not only the fruit yield but also their quality, as was previously reported for *Rhizobium* in other raw consumed vegetables [7].

Nevertheless, the main characteristic of functional fruits is their high content of bioactive compounds, some of them increasing when plants are biofertilized with *Rhizobium* [42]. In strawberry fruits the main bioactive compound is the vitamin C [18] and as far as we are aware there are no data about the positive effects of bacterial biofertilizers on the content of this vitamin. Although increases in the vitamin C contents of strawberry fruits have been reported after coinoculation with mycorrhiza and bacteria, this effect is not due to the bacteria inoculated [41]. Then this is the first work in which it is showed a significantly higher increase in vitamin C levels in strawberry fruits after inoculation with plant probiotic bacteria.

It may thus be concluded that bacterial plant probiotics increase not only the growth, yield and quality of plants, but also that they are a tool to produce highly functional foods hence benefiting the human health in a dual way, namely the replacement of chemical fertilizers by biofertilizers and the increase in bioactive compounds in plant foods.
